# Atypical Presentation in X-linked Immunodeficiency With Magnesium Defect, Epstein-Barr Virus (EBV) Infection, and Neoplasia (XMEN) Disease: A Case Report and Review of Emerging Therapies

**DOI:** 10.7759/cureus.91193

**Published:** 2025-08-28

**Authors:** Micah Madsen, Richard Sidlow

**Affiliations:** 1 Genetics, Ben Gurion University of the Negev, Beersheba, ISR; 2 Medical Genetics and Metabolism, Valley Children's Hospital, Madera, USA

**Keywords:** immunodeficiency disease, magnesium supplementation, magt1 mutation, pediatric immunodeficiency, xmen disease, xmen disease therapies

## Abstract

X-linked immunodeficiency with magnesium defect, Epstein-Barr virus infection, and neoplasia (XMEN) is a rare primary immunodeficiency caused by MAGT1 mutations. These mutations impair N-linked glycosylation and magnesium-dependent signaling in T cells, disrupting immune surveillance. XMEN typically presents with chronic Epstein-Barr virus (EBV) viremia, lymphoproliferative disease, and viral infections, although phenotypic variability is increasingly recognized. A seven-year-old male presented at age five with bilateral conjunctival hemorrhages and petechiae and was found to have severe thrombocytopenia (platelet count: 4,000/µL). Initial treatment for presumed idiopathic thrombocytopenia (ITP) with idiopathic thrombocytopenic purpura (IVIG) and corticosteroids yielded minimal improvement. Bone marrow biopsy excluded malignancy, and he was maintained on romiplostim with variable platelet recovery. Two years later, he was evaluated by genetics due to persistent ITP, steroid side effects, and disseminated molluscum contagiosum. Genetic testing revealed a pathogenic hemizygous deletion of exons 2-10 in MAGT1, confirmed by chromosomal microarray. This deletion is consistent with XMEN disease. Family testing showed no mutation in his two full brothers, suggesting a de novo variant. Despite low platelet counts and elevated liver enzymes, he remained free of systemic infections and EBV-related complications. Magnesium supplementation resulted in moderate improvement of molluscum contagiosum lesions, but thrombocytopenia persisted. This case illustrates an atypical presentation of XMEN disease, with isolated thrombocytopenia and cutaneous viral infection in the absence of EBV viremia or lymphoproliferative disease. It supports a broader clinical spectrum for XMEN and underscores the need for continued research into genotype-phenotype correlations and targeted therapeutic approaches.

## Introduction

X-linked immunodeficiency with Mg2+ defect, Epstein-Barr virus (EBV) infection, and neoplasia (XMEN) disease is a rare combined immunodeficiency. It is caused by a loss-of-function mutation in the magnesium transporter 1 (MAGT1)gene found on the X chromosome [1]. Ravell et al. were among the first to demonstrate that mutations in MAGT1 are responsible for defective glycosylation of several important lymphocytic proteins, including the T-cell receptor (TCR) alpha and beta chains, the co-stimulatory molecules CD28 and CD70, and the activator receptor natural killer (NK) group 2, member D (NKG2D) [2,3]. These immune proteins play a key role in immune regulation, and impaired glycosylation results in their degradation and decreased surface expression, particularly on CD8+ and NK cells [3]. Impaired NKG2D, in particular, is critical for the recognition and killing of virally infected cells. The decreased surface expression disrupts the patient’s cytotoxic responses against various pathogens, notably, EBV [2,4].

In addition to its role in glycosylation, MAGT1 also regulates a transient influx of Mg2+ into T-cells, which is essential for proper downstream signaling of the TCR [2]. MAGT1 mutations result in a reduced influx of Mg2+ into T-cells, leading to disrupted magnesium-dependent enzymatic and TCR signaling activities. Studies have shown that MAGT1 mutations particularly affect CD8+ T cells, thereby reducing their activation, proliferation, and cytotoxic activity [3].

In the cases reported, the disease appears to have complete penetrance with variable expressivity, resulting in a range of clinical manifestations [5]. The most common presentation of XMEN disease is an increased susceptibility to EBV infection and EBV-associated lymphoproliferation [2]. In addition, patients frequently exhibit a range of multi-system abnormalities, such as sinopulmonary and ear infections, upper and lower respiratory tract infections, mouth ulcerations, hepatosplenomegaly, skin warts, and hematopoietic neoplasia [5]. They are prone to various acute viral infections, including varicella zoster virus, molluscum contagiosum, and herpes simplex virus [2]. Laboratory findings are variable but often include elevated liver enzymes, hypogammaglobulinemia, thrombocytopenia, neutropenia, decreased CD8+ cell levels, and low levels of IgG and IgA [1,5]. Neuropsychiatric symptoms, though less common and poorly understood, are increasingly recognized [1]. Recent case reports have emphasized a neurological aspect of the disease, with certain neuropsychiatric symptoms appearing even during adulthood [1,6,7].

We present a case of a young patient with XMEN disease, whose only clinical features were isolated thrombocytopenia, molluscum contagiosum, and mild transaminasemia.

## Case presentation

The patient is a seven-year-old male initially referred at age five for bilateral conjunctival hemorrhages, serosanguinous discharge, and periorbital swelling. At this time, his vision and red reflex were normal. Physical examination revealed petechiae and bruising on the torso, arms, and legs. Laboratory tests showed severe thrombocytopenia with a platelet count of 4,000/µL (neutrophils: 168-472 x 10^3^/µL). Computed tomography of the brain was unremarkable. He was diagnosed with idiopathic thrombocytopenia (ITP) and treated with intravenous immunoglobulin, but follow-up platelet counts three days later showed only a slight increase to 6,000/µL. A bone marrow biopsy ruled out leukemia, and he was subsequently treated with high-dose methylprednisolone, achieving a platelet count of 14,000/µL upon discharge.

Two weeks later, he returned with active bleeding, hematuria, and anemia, requiring intravenous methylprednisolone and a red blood cell transfusion. He was discharged with a platelet count of 21,000/mcL and started and maintained on weekly subcutaneous romiplostim, which was effective in preventing thrombocytopenic crises but failed to consistently maintain platelet counts within normal limits.

His initial encounter with genetics was at age seven. His physical examination was normal, except for stigmata consistent with chronic steroid use and disseminated molluscum contagiosum. He was in foster care and was seen with his two full brothers, who were under the care of the same foster parents. Two sisters and a fraternal twin brother and sister were known to exist but were of unknown paternity. No pregnancy, delivery, developmental, or family history was available.

**Figure 1:**
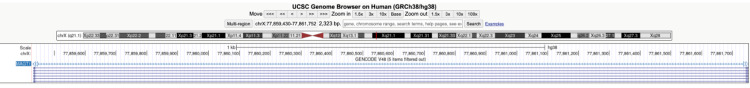
Genomic location of the MAGT1 gene on the X chromosome Genomic position of the MAGT1 gene visualized using the UCSC Genome Browser (GRCh37/hg19 assembly). The image shows the location on Xq21.1 with transcript isoforms and conservation tracks. Displaying the precise genomic context emphasizes the gene’s location on the X chromosome, consistent with the X-linked inheritance pattern, and illustrates the high degree of evolutionary conservation, underscoring the functional importance of MAGT1 in immune regulation. Image generated from genome.ucsc.edu [8].

Upon chart review, results from a primary immunodeficiency panel (Invitae; San Francisco, CA) performed at age five revealed a hemizygous and pathogenic deletion of exons 2-10 in the MAGT1 gene. This finding was confirmed by chromosomal microarray analysis (GeneDx; Gaithersburg, MD), which identified a 71 kb deletion-arr(GRCh37) Xq21.1(77065542_77136498)x0 (NM_032121.5)-corresponding to the loss of exons 2-10 in MAGT1, consistent with XMEN disease (see Figure 1). Additionally, the microarray showed runs of homozygosity encompassing 2.21% of the autosomal genome. Due to its X-linked inheritance pattern, the patient’s two biological brothers underwent genetic testing, which confirmed that neither carried the MAGT1 pathological variant, arguing for its de novo origin in our patient.

Platelet counts remained consistently low throughout the first month following his XMEN diagnosis, ranging from a nadir of 9,000/μL to a peak of 46,000/μL. Liver enzymes were elevated during the first month, with aspartate transaminase decreasing from a peak of 104 U/L to 27 U/L by month’s end, and alanine transaminase declining from 224 U/L to 45 U/L. Despite his transaminase elevation, no symptoms of hepatitis or hepatic failure were noted. Treatment with romiplostim and prednisone was continued, resulting in only minimal improvement in platelet counts; however, bleeding symptoms remained well-controlled. Magnesium supplementation was added with moderate improvement of his molluscum contagiosum lesions.

## Discussion

In their review of presentations of XMEN disease, Ravell et al. found that mild thrombocytopenia is present in up to one-third of all patients [5]. Interestingly, in EBV-naive patients, severe ITP commonly manifests as the presenting symptom, which highlights the need to examine how MAGT1 mutations impact platelet function and levels [5].

Recent studies by Kauskot et al. and del Pino Molina et al. demonstrated that magnesium plays an important role in platelet function, and MAGT1 is believed to help regulate magnesium levels in platelets [9,10]. Their findings showed that MAGT1 is crucial for N-glycosylation of platelet receptors, a key step in platelet activation. MAGT1 variants led to altered glycosylation of platelet receptors, contributing to platelet dysfunction [10]. This led to not only impaired platelet aggregation, but also defective integrin activation, disrupted calcium mobilization, and reduced protein kinase C activity [9]. These findings suggest that XMEN is not only a congenital glycosylation disorder that causes immune cell deficiency but also platelet function abnormalities. However, the cause of reduced platelet counts to the point of causing severe ITP in select XMEN patients currently remains unclear.

To date, no curative therapy is available for XMEN disease. Early in vitro studies were promising regarding magnesium supplementation in partially restoring NKG2D expression, particularly in patients who were MAGT1 deficient [2,11,12]. Iyengar et al. reported that magnesium supplementation was associated with a reduction in molluscum lesions within six months, along with increased expression of NKGD2D receptors on NK and CD8+ T cells [12]. This aligns with our patient’s case, in which magnesium supplementation led to a reduction in molluscum contagiosum lesions, but did not result in any improvement in thrombocytopenia. The overall effect of magnesium, however, may differ among XMEN patients. A recent placebo-controlled, double-blind study in four patients with XMEN disease found no beneficial effect of magnesium supplementation in aiding NKG2D expression [13]. This underscores the need for further research to fully determine the efficacy of magnesium supplementation.

In recent years, several innovative treatments have been investigated. Allogeneic hematopoietic stem cell transplantation has been suggested as a potential cure; however, reports in XMEN patients have either been inconclusive or associated with high mortality rates [9,14].

Ding et al. [15] studied the activation of tumor candidate suppressor 3 (TUSC3) as a possible therapeutic therapy for XMEN patients. Using a MAGT1 knockout NK cell line, they developed an in vitro model to replicate the lymphocytic phenotypes derived from XMEN patients. They found that the epigenetic drugs decitabine and panobinostat restored TUSC3 and NKG2D expression, which ultimately rescued lymphocytic cytotoxic function and improved liver abnormalities [15]. These findings suggest that epigenetic therapy could serve as a potential treatment for XMEN patients, given that these drugs exhibit manageable side effects and a satisfactory safety profile [15].

Taking a different approach, Brault et al. used MAGT1 mRNA electroporation to correct autologous lymphocytes from XMEN patients [16]. Their results showed that NKG2D expression was restored in CD8+ T and NK cells just one to two days after electroporation, with expression maintained at ~50% up to two weeks later. This mRNA correction not only restored NK cell cytotoxicity, but also the NKG2D receptor expression and function were unaffected by cryopreservation. They proposed repeated infusions of MAGT1 mRNA-corrected autologous CD8+ and NK cells as a potential short-term therapy for XMEN patients [16].

## Conclusions

This case highlights an atypical presentation of XMEN disease in an EBV-naive patient, characterized by severe thrombocytopenia and molluscum contagiosum. While XMEN is classically associated with EBV-related lymphoproliferation, our findings support expanding the clinical spectrum to include isolated hematologic and dermatologic manifestations, particularly in early childhood.

Recent insights into MAGT1’s role in platelet glycosylation and immune regulation provide a plausible explanation for these findings. Genetic testing remains essential for diagnosis, especially in cases of refractory ITP with multisystem involvement. Although magnesium supplementation showed partial improvement in skin lesions, its therapeutic benefit appears limited and variable. Emerging therapies such as epigenetic modulation and mRNA correction offer promising future directions but remain experimental.
